# Approaches to Understanding and Addressing Treatment-Resistant Depression: A Scoping Review

**DOI:** 10.1155/2012/469680

**Published:** 2012-04-18

**Authors:** Emily Jenkins, Elliot M. Goldner

**Affiliations:** ^1^School of Nursing, University of BC, Vancouver, British Columbia, Canada V6T 2B5; ^2^Centre for Applied Research in Mental Health and Addiction, Faculty of Health Sciences, Simon Fraser University, Vancouver, BC, Canada V6B 5K3

## Abstract

Treatment-resistant depression is associated with significant disability and, due to its high prevalence, results in substantive economic and societal burden at a population level. The objective of this study is to synthesize extant literature on approaches currently being applied to understand and address this condition. It is hoped that the findings can be used to inform practitioners and guide future research. A scoping review of the scientific literature was conducted with findings categorized and charted by underlying research paradigm. Currently, the vast majority of research stems from a biological paradigm (81%). Research on treatment-resistant depression would benefit from a broadened field of study. Given that multiple etiological mechanisms likely contribute to treatment-resistant depression and current efforts at prevention and treatment have substantial room for improvement, an expanded research agenda could more effectively address this significant public health issue.

## 1. Introduction

Of the population receiving treatment for depression, a large proportion is not responding adequately to current treatment approaches, with an estimated 50–70% described as treatment resistant [1, 2]. A consistent and commonly accepted definition for treatment-resistant depression (TRD) remains elusive. Greden [1] argues that remission should be the ultimate goal of treatment and absence of remission should be the criterion used for determining the presence of TRD. Berlim and Turecki have noted over 10 different definitions for TRD in the specialized literature [3, 4]. However, in a systematic review of randomized trials (*n* = 47), they report a general consensus within the literature defining clinically significant TRD as “an episode of major depression (that) has not improved after at least two adequate trials of different classes of (antidepressants)” (*n* = 26) [4, page 703]. The authors further argue that TRD should be viewed on a continuum ranging from partial response to complete treatment resistance as opposed to an all or nothing phenomenon. They point out that an inadequate response to treatment should constitute failure to reach remission, and that future studies should employ this criterion as the “gold standard outcome,” a conclusion that parallels Greden's work [1].

Fava defines TRD as a failure to achieve remission following an adequate trial of antidepressant therapy [5]. This definition differs from that proposed by Berlim and Turecki in that it does not necessitate* two* adequate trials of antidepressant treatment [3, 4]. Like other TRD researchers [1, 3, 4], Fava stresses the importance of remission as the treatment goal, noting that individuals with residual depressive symptoms show poorer long-term outcomes and increased relapse risk [6], a finding supported by other research in the field [7, 8].

Given the inconsistency in definition, it is difficult to accurately measure the prevalence or disease burden associated with TRD. However, despite the conceptual limitations, the burden of illness associated with TRD is considered to be substantial. TRD is posited to be responsible for the greatest burden associated with depressive disorders [1]. The suffering and disability associated with chronic, unremitting depressive illness is profound. In an effort to better quantify the economic burden associated with TRD, Greenberg and colleagues conducted a study of an employed population to identify the differential health care costs associated with nonresistant depression, and TRD [9]. Health care costs associated with TRD employees were found to be more than double the costs associated with employees with nonresistant depression and almost quadruple the costs associated with employees from a random sample. Greden argues that the disease burden associated with TRD is unacceptably large and represents a significant public health issue requiring a paradigm shift towards prevention, early intervention, and adequate treatment [1].

The objective of this scoping review was to examine the range of approaches being applied to understand and address TRD in an effort to guide research and practice in this area. A scoping review is a relatively new research approach which allows for quick and systematic identification of the breadth of literature in a subject area of interest. This methodology, which aims to illuminate the *scope* or extent of literature in a particular area, can be contrasted with systematic reviews which have a narrower focus and seek to answer well-defined research questions from the available literature, while also encompassing a component to determine the quality of research reviewed. The York scoping review methodology outlined by Arksey and O'Malley [10] describes scoping reviews as having at least four potential functions: to map the current state of literature in an area of interest, to determine the value or feasibility of conducting a systematic review, to summarize and disseminate research findings to an audience (e.g., policy makers, clinicians, consumers, etc.), and finally, to identify gaps where further research is required. The scoping review process involves five essential steps: (1) determining the research question, (2) identifying relevant studies, (3) selecting studies, (4) charting data, and (5) collating, summarizing, and reporting results [10]. This scoping review utilized the York framework to survey the literature to identify the current state of evidence surrounding the approaches and paradigms being utilized to understand and address TRD.

## 2. Materials and Methods

A systematic search of the literature was undertaken utilizing six electronic databases (Academic Search Premier, CINAHL, Medline, PsycArticles, PsychInfo, and Health Source). Keywords to identify literature related to TRD included: “treatment-resistant and depression,” “refractory and depression,” “therapy-resistant and depression,” “treatment-resistant and depression and social,” “treatment-resistant and depression and nonpharmacological,” “treatment-resistant, and depression and novel,” “medication-resistant and depression,” “treatment-resistant and depression and burden,” “treatment-resistant and depression and public health.” Resulting titles (*n* = 2392) were scanned to identify articles that met the inclusion criteria which included research articles related to unipolar TRD amongst the adult population (ages 18 and 65) with English language abstracts available, and publication dates from 2005–2010. Letters to the editor and responses to articles were excluded. This process produced a total of 345 articles that were exported to reference management database, Mendeley, for analysis. These papers were then hand-searched and the data charted by theme. Thematic classification was carried out by the lead author, with decisions reviewed by second author. Where discrepancies in classification existed, the article in question was discussed and agreement reached between authors. These themes inform the structure of the following results.

## 3. Results

The vast majority of the literature (*n* = 277; 80%) focused on treatment with much less attention directed toward identifying the causal factors that contribute to treatment resistance (*n* = 33; 10%) or discussing conceptualizations of the illness, current treatment trends, definitional and treatment issues, and identifying research gaps (*n* = 36; 10%). The literature identified through the scoping review was classified thematically by underlying paradigm as biological (*n* = 281; 81%), psychological (*n* = 11; 3%), social (*n* = 2; 1%), multiple or combined paradigms (e.g., biological and psychological therapies used simultaneously, *n* = 6; 2%), and other (*n* = 45; 13%). We describe findings classified by each paradigm or combination below.

### 3.1. Biological Paradigm

Of the articles identified, 81% (*n* = 281) fell within the biological paradigm. The literature in this theme was further classified by focus as pharmacotherapy (*n*  = 145), neurostimulation, and neurosurgical interventions (e.g., repetitive transcranial magnetic stimulation (rTMS), vagus nerve stimulation, deep brain stimulation, electroconvulsive therapy, and neurosurgery, *n* = 104), biologically based causal mechanisms (*n*  = 30), and other (*n* = 2).

The psychopharmacology literature focuses largely on determining efficacious augmentation strategies for those who have not achieved remission following antidepressant monotherapy. Augmentation is a common approach employed in the treatment of TRD and is endorsed by various TRD treatment staging models including the Thase and Rush Model of Staging Treatment Resistance, the Massachusetts General Hospital Staging Method, and the European Staging Method [3, 5]. While many of the articles represent reviews or discussions surrounding augmentation strategies, others provide evidence of the efficacy of specific augmentation combinations. For example, Anderson et al. [11] conducted a small (*n* = 24) open-label trial to explore the efficacy of atypical antipsychotics (i.e., quetiapine) as an augmentation strategy for patients diagnosed with TRD who were currently taking a monoamine reuptake inhibitor. These authors conclude that quetiapine augmentation may be helpful for patients with TRD, but acknowledge that placebo-controlled studies with larger sample sizes are required. In fact, the absence of large, controlled trials of specific augmentation strategies is a concern identified in a review by Carvalho et al. [12]. These authors conducted a literature review of augmentation strategies and point out a current shortcoming within the literature that although there are numerous studies of different pharmaceutical augmentation strategies, to date, few represent large, controlled trials in which clinicians can have confidence in the findings. The types of medications studied for use in augmentation therapy of TRD include mood stabilizers (e.g., lithium, lamotrigine), monoamine oxidase inhibitors, tricyclic antidepressants, thyroid hormones (e.g., T3), vitamin supplements (e.g., zinc), antipsychotics (e.g., quetiapine, olanzapine, risperidone), nicotinic antagonists, antibiotics (e.g., D-cycloserine), psychostimulants (e.g., methylphenidate), opiates (e.g., buprenorphine), and anaesthetics (e.g., ketamine).

Like the pharmacotherapy literature, much of the literature on other biological therapies focuses on determining the efficacy of a specific treatment, or combination of treatments, with a small body of research exploring this intervention on a more micro level seeking to identify the pathophysiologic changes associated with these interventions. The efficacy studies conducted on rTMS report primarily promising results. In a systematic review and meta-analysis of rTMS for TRD, Lam and colleagues [13] identified 24 controlled trials assessing the efficacy of active versus sham rTMS and conclude that rTMS demonstrates positive results. However, the authors argue that further studies are required given limitations resulting from relatively small effect sizes, short treatment periods (i.e., 1–4 weeks), and lack of systematic followup after completion of the intervention period. Taking a more microlevel approach to exploring rTMS, Kito et al. [14–17] have undertaken a number of studies exploring the brain regions associated with rTMS and antidepressant response. For example, in a 2008 study, Kito and colleagues [14] investigated the neuroanatomical changes associated with the application of high frequency rTMS. Using single-photon emission computed tomography, the researchers produced brain imaging that lead them to conclude that the antidepressant effects of rTMS are, in part, a result of changes in the function of both the left dorsolateral prefrontal cortex and the limbic-paralimbic regions of the brain.

Similarly, the studies on vagus nerve stimulation, deep brain stimulation, electroconvulsive therapy, and neurosurgery were largely focused on determining the safety and efficacy of these treatment approaches. A small number of the studies that addressed these nonpharmacological biological approaches also looked at the efficacy of combining these treatments with each other (e.g., [18, 19]). For example, Burke and Husain [18] explored the implications of combined vagus nerve stimulation and electroconvulsive therapy. The authors conclude that this novel approach to addressing TRD is safe; the two therapies can be used together. Further, the authors suggest that both of these interventions can be employed to address different clinical manifestations of TRD: vagus nerve stimulation for treatment of chronic depressive symptoms and electroconvulsive therapy for worsening depressive symptoms and maintenance. Many of the studies categorized as neurostimulation and neurosurgical interventions demonstrated inconsistent outcomes, had small sample sizes, or were limited to brief follow-up periods.

The majority of studies classified within the biological paradigm addressed therapeutic intervention trials in humans (*n* = 251). However, a number of other studies (*n* = 30) sought to identify the biologically based causal factors associated with TRD. These investigations included human brain imaging studies as well as research seeking to detect various biological markers or mechanisms involved in the experience of TRD (e.g., lower plasma levels of Coenzyme Q10 [20], lower cerebral spinal fluid levels of substance P [21], impaired glucocorticoid receptor function [22]). In 2009, Juruena and colleagues [23] published the results of a prospective study exploring the functioning of the hypothalamic-pituitary-adrenal (HPA) axis in patients with depression. The researchers were able to demonstrate that patients with depression experience HPA axis overactivity and, further, that the negative feedback system that typically controls HPA axis activity is impaired in patients experiencing TRD. Given these findings, the authors suggest that neuroendocrine dysfunction is a reliable marker for poor treatment response in patients presenting with depression.

### 3.2. Psychological Paradigm

Approximately 3% (*n* = 11) of the articles identified through this scoping review could be classified as stemming from a psychological paradigm. This literature consisted predominantly of studies exploring the application of cognitive behavioural therapy in the treatment of TRD (*n* = 7). However, there is a limited literature on more novel psychological approaches including mindfulness and dialectical behavioural therapy. Many of the articles falling into this theme are discussion papers as opposed to empirical studies. However, the limited literature that does provide data on the efficacy of psychotherapy for TRD reports favourable outcomes. For example, Watkins and colleagues [24] conducted a study of rumination-focussed cognitive behavioural therapy. Rumination is identified as a key factor in the onset and maintenance of depressive symptomology. In this small study (*n* = 14), patients with TRD were provided with up to 12 sessions of one-on-one cognitive behavioural therapy. The authors report that the intervention resulted in significant reductions in symptomology, with 50% of their participants achieving remission. In another study exploring cognitive behavioural therapy for patients experiencing TRD, the researchers took a group therapy approach, which also demonstrated positive outcomes. Matsunaga et al. [25] explored the effectiveness of group-based cognitive behavioural therapy addressing psychosocial functioning and depressive symptomology amongst patients with “mild” TRD. Participants in this study (*n* = 38) attended 12 sessions of cognitive behavioural group therapy over a 12-week period. Following the intervention, participants demonstrated significant reductions in depressive symptomology and enhanced psychosocial functioning. Further, the intervention appeared to produce sustainable improvements; participants who completed the 12-month followup measures (*n* = 20) continued to demonstrate improved psychosocial and mood outcomes.

### 3.3. Social Paradigm

This scoping review identified only two articles classified as stemming from a social paradigm. This small literature describes various social factors found to be associated with TRD including: poor baseline financial status, rural area of residence (which was proposed to influence TRD through lower levels of neighbourhood and personal socioeconomic status, and a greater likelihood of receiving less than adequate drug treatment), lower levels of psychosocial functioning [26], and exposure to serious life events (specifically, job loss, and financial distress) [27]. However, even within this limited literature, there are inconsistent findings. For example, while Amital and colleagues [27] report specific serious life events to be associated with TRD, Viinamäki et al. [26] found no relationship between serious life events and this illness.

### 3.4. Multiple Paradigms

In addition to the literature that could be clearly classified as biological, psychological or social in paradigm, there was also a very limited research exploring a combination of these approaches (*n* = 6). This literature includes five primary studies and one discussion paper, all of which focus on the effectiveness of combining medications with psychotherapy (i.e., cognitive behavioural therapy or interpersonal therapy). This literature indicates that utilizing a combination of biological and psychological approaches results in superior treatment outcomes as compared to single mode approaches. Schramm and colleagues [28] conducted a study on an inpatient psychiatric unit in which they randomized patients (*n* = 45) to either a treatment arm, in which participants received medication therapy combined with interpersonal psychotherapy (utilizing a combination of individual and group-therapy formats), or a control arm, in which participants received medication therapy and clinical management (i.e., usual care). Following the five-week intervention, patients who received interpersonal psychotherapy in combination with pharmacotherapy showed statistically significant improvement over those who received clinical management in combination with medication therapy (remission rates amongst the psychotherapy group were 67% versus 32% among the clinical management group). Further, at 12-month follow up, only 7% of the pharmacotherapy/psychotherapy participants had experienced a depressive relapse compared with 25% of the pharmacotherapy/clinical management group participants. Although the findings of this study suggest that interpersonal psychotherapy leads to enhanced treatment outcomes over usual care, the authors note limitations to their study including unequal levels of therapeutic attention between participant groups and the inpatient status of their participants (limiting generalizability of the intervention findings to this patient subgroup). In another study exploring the combination of psychotherapy and pharmacotherapy, Wiles and colleagues [29] conducted a feasibility study of cognitive behavioural therapy in combination with pharmacotherapy for primary care patients experiencing TRD, noting that to date, no randomized controlled trials of this type of intervention have been conducted. The researchers report that their study demonstrates that primary care patients with TRD are agreeable to participating in psychotherapy in combination with pharmacotherapy and advocate for large-scale randomized controlled trials to demonstrate the effectiveness of this multimodal approach.

### 3.5. Other

In addition to the papers that we were able to categorize as drawing from a particular paradigm, there were a number of papers (*n* = 45) which did not fit within this classification system. These manuscripts represent discussion papers surrounding various topics related to TRD including: definitional concerns (e.g., [3, 4, 30]), current treatment trends (e.g., [31, 32]), overviews of the illness and suggested “next steps” (e.g., [31]), descriptions of the work of select large-scale studies (e.g, [33, 34]), and clinical issues and treatment staging models (e.g., [35, 36]).

## 4. Discussion

Despite acknowledgement that “depression is the product of a complex interaction between biological, psychological and social elements” [37, page 5], this scoping review found that the study of TRD has been focused primarily on biologically based treatments.

### 4.1. Biological Dominance

The biological paradigm has dominated mental health research and practice for the last several decades [38]; resulting in a situation Cohen describes as biological reductionism—a skewed view of mental health and illness that does not adequately consider the social or psychological aetiology of these disorders [39]. The preponderance of the biological approach is apparent in the TRD research literature and appears to influence the proposed definitions, treatment staging, treatment approaches, and types of research being conducted. This is exemplified by the currently proposed definitions for TRD which are based on failure to respond to antidepressant medication. This produces a tautology in which the presence or absence of TRD is based solely on response to a biological treatment. These proposed definitions fail to adequately acknowledge the complexity of this illness and the limitations of this approach.

Pharmacotherapy was a central focus of the research literature, with 52% of the articles within the biological paradigm reporting on this treatment approach. It is possible that the overreliance on pharmacotherapy as an approach to TRD may be limiting the effectiveness of current treatment efforts.

### 4.2. Seeking a Balanced Approach

A significant gap in the current literature is research seeking to identify the causal factors associated with TRD from a social or psychological perspective. Without epidemiological or social science studies addressing this knowledge gap, we lack an understanding of *who* is at risk for TRD; evidence that is necessary to inform the development of more effective prevention and treatment strategies. However, from the research that has been conducted in this area, it is clear that a more balanced approach to understanding and addressing TRD is needed; one that acknowledges and targets the complex and diverse aetiology of this illness. For example, within the limited research exploring combination approaches to TRD, the findings indicate that broader approaches result in enhanced treatment outcomes [40, 41].

Given the multifaceted causal mechanisms underlying TRD, we hypothesize that the most effective treatment approaches would be equally complex and include interventions that address the diverse factors contributing to this debilitating illness. Interestingly, interdisciplinary and multimodal approaches to TRD remain novel, as evidenced by the very limited work identified in this area. For example, although psychotherapy has been established in the clinical guidelines literature as an effective, evidence-based treatment for depression [42]; research exploring the utility of psychological approaches for TRD remains extremely limited.

Current efforts to address TRD represent a “downstream approach”—seeking to address the problem once it is already causing substantial distress and disability. The downstream approach is common in health care, but is not necessarily the most effective strategy for reducing incidence and prevalence of disorder. With the growing prevalence of depression and TRD, and the resulting burdens, research identifying appropriate “upstream approaches” is needed. Upstream approaches to health target the root causes of the issue and help to prevent incidence of illness. Research identifying the various social factors contributing to this disorder is necessary. This would allow for interventions and policies that address the social determinants of depression and foster mental health. The limited research that is available indicates that social factors are predictive of treatment-resistance [26]. One could imagine that if members of the TRD population are experiencing adverse life situations related to socioeconomic status (e.g., poor working conditions, unemployment, low levels of education, etc.), gender (e.g., violence, stress related to competing life roles, sexism, etc.), or other social factors (e.g., loss of a loved one, financial insecurity, etc.) that attention to these issues may contribute to improved health outcomes, and better prevention and treatment approaches in the future.

Our lack of knowledge surrounding the various causal factors associated with this illness should be of significant concern to policy makers, researchers, health professionals, and the general public. Without a better understanding of the disorder, the health, social, and economic burdens associated with TRD will continue to constitute a substantive public health problem. Further research encompassing broader perspectives and approaches is necessary if we hope to make progress in reducing the incidence and prevalence of TRD.

### 4.3. Definitional Difficulties and Skewed Staging Models

In addition to the problems stemming from an overemphasis on biological paradigms, a significant barrier to better addressing TRD is the lack of a universally accepted definition. The absence of a consistent definition of TRD has been problematic in the literature in that it has led to difficulties in accurately and consistently measuring related constructs. However, these issues may actually serve as an opportunity for researchers, clinicians, and policy makers to come together in an effort to create necessary change. Efforts to expand the definition of TRD to more accurately reflect the experience and to capture therapies beyond the biological paradigm are needed.

In addition to issues surrounding the biological dominance of current research and treatment approaches, the lack of research evidence exploring the causal factors associated with TRD, and the inconsistent definitions proposed for the illness, there are challenges associated with the detrimentally narrow focus within the treatment staging models employed. Fava acknowledges the “pharmaco-centric” approach to TRD, and the need to incorporate other evidence-based therapies, including psychotherapy, into the depression treatment approach [5]. However, despite this claim, nonbiological therapies do not appear in the most common treatment staging models [43]. Future research and practice guidelines must acknowledge the important role of non-pharmacologic interventions in the treatment of depression, and incorporate these approaches into a widely accepted diagnostic definition and treatment algorithm for TRD.

## 5. Conclusions

Current treatment approaches to depression are not effective in producing remission in a large proportion of those affected by this illness, resulting in a high prevalence of TRD, a form of depression which produces significant disease burden. Further, despite evidence that depression results from the interaction of biological, psychological, and social factors, the scientific literature addressing TRD remains unbalanced, with little research focusing on the psychological or social aspects of the illness. This scoping review highlights the need to expand the scope of research being conducted in order to decrease the substantial burden associated with TRD and improve health outcomes for those experiencing this debilitating illness.
